# Psychosocial determinants of job satisfaction: the case of employees of a Tunisian electricity and gas company

**DOI:** 10.1192/j.eurpsy.2022.1612

**Published:** 2022-09-01

**Authors:** N. Rmadi, N. Kammoun, R. Masmoudi, N. Kotti, J. Masmoudi, K. Hammami, M.L. Masmoudi, M. Hajjaji

**Affiliations:** 1 HEDI CHAKER hospital, Department Of Occupational Medicine, SFAX, Tunisia; 2 occupational health and safety institute, Department Of Occupational Medicine, Tunis, Tunisia; 3 Hospital university of HEDI CHAKER, Psychiatry A Department, Sfax, Tunisia

**Keywords:** job satisfaction, occupational stress

## Abstract

**Introduction:**

Over the past decades, a growing body of evidence has demonstrated the impact of the psychosocial work environment on workers’ health, safety and wellbeing. These factors may also affect employees ’job satisfaction.

**Objectives:**

To explore psychosocial determinants of job satisfaction among workers in a Tunisian electricity and gas company.

**Methods:**

A cross-sectional survey was conducted among male workers in a Tunisian electricity and gas company. The Copenhagen Psychosocial Questionnaire (COPSOQ), the Job Content Questionnaire and the general health questionnaire (GHQ12) were used to assess psychosocial risk factors at work. A principal component analysis (PCA) was used to assess correlations between instruments ’scores. Multiple linear regression analysis was applied to explore the specific factors associated with job satisfaction. Data were analysed using R software.

**Results:**

A total of 83 workers participated in the survey (the age range: 21-60 years). Job satisfaction score varied from 0 to 100% with a mean of 73.09 %. In the PCA, job satisfaction had a positive correlation with high social support and a negative one with work-family conflicts, a high psychological demand, stress, burnout and quantitative demands. In multivariate analysis, factors negatively associated with job satisfaction were: age, stress and low social support. In contrast, seniority was positively associated with job satisfaction.

**Conclusions:**

Job satisfaction is deeply influenced by the psychosocial work environment. Therefore, it is necessary to provide supervision, communication, and social support for these workers to increase or maintain a high level of job satisfaction.

**Disclosure:**

No significant relationships.

